# Effect of febuxostat on blood pressure in hyperuricemic patients: A protocol for a systematic review and meta-analysis

**DOI:** 10.1097/MD.0000000000020673

**Published:** 2020-06-12

**Authors:** Jia Yao, Xiaoyan Shi, Simin Fan, Yang Gao, Hengchang Hu, PanPan Wang, Qiu Chen

**Affiliations:** School of Clinical Medicine, Chengdu University of Traditional Chinese Medicine, Chengdu, Sichuan Province, P.R. China.

**Keywords:** febuxostat, blood pressure, hypertension, chronic kidney disease, meta-analysis

## Abstract

**Background:**

Increasing evidence connects serum uric acid (sUA) with hypertension. Previous studies on the efficacy of febuxostat on blood pressure (BP) in hyperuricemic patients have provided conflicting results. Thus, we aim to perform a systematic review and meta-analysis to investigate the efficacy of febuxostat on BP.

**Methods:**

Five electronic databases (included The Cochrane Library, MEDLINE, Embase, Web of Science, and Cochrane Central Register of Controlled Trials) will be searched. Randomized controlled trials will be included if they recruited hyperuricemic participants for assessing the effect of febuxostat on BP versus control (placebo, no treatment, and other therapeutic agents). The primary outcome will be BP, secondary outcomes will be sUA, serum creatinine, and estimated glomerular filtration rate. Relevant literature search, data extraction, and quality assessment will be performed by 2 researchers independently, and the third researcher will be involved in a discussion for any disagreements. All analyses will be performed based on the Cochrane Handbook for Systematic Reviews of Interventions. Stata 12.0 software will be used for statistical analysis. The effect size of dichotomous data will be measured using the odds ratio , and the effect size of continuous data will be measured using the standardized mean difference. And 95% confidence intervals will be calculated. Heterogeneity will be tested by *χ*^2^-based Cochran Q statistic and *I*^2^ statistic. Sensitivity analysis and subgroup analysis will be used to observe changes in the pooled effect size and heterogeneity between included studies, to assess the reliability and stability of the pooled results. The funnel plot and Egger's and Begg's tests will be used to judge publication bias, and the trim and fill method will be used to correct the funnel asymmetry caused by publication bias. *P* < .05 will be considered to indicate a statistically significant result.

**Results:**

This systematic review and meta-analysis will be to assess the efficacy of febuxostat on BP.

**Conclusions:**

Our findings will show the effect of febuxostat on BP in hyperuricemic patients. And such a study may find a new therapeutic option for hypertensive patients and assist clinicians and health professionals make clinical decisions.

**Ethics and dissemination:**

This study is a protocol for systematic review and meta-analysis of the effect of febuxostat on BP in hypertensive patients. This systematic review and meta-analysis will be published in a journal and disseminated in print by peer-review.

**INPLASY registration number:**

INPLASY202050031.

## 1 Introduction

Uric acid (UA) is the end product of the metabolism of purine compounds. Hyperuricemia is the critical precursor to gout, which can result from excessive UA production, reduced excretion, or both. Apart from gout, hyperuricemia is proved to be connected with many other diseases including hypertension and renal disease.^[1–6]^

Large epidemiological studies evaluating the relationship between hyperuricemia and hypertension proved that hyperuricemia was an independent risk factor for hypertension.^[7,8]^ And a meta-analysis of 25 studies (n = 97,824) further proved this.^[9]^ Besides, a large number of studies have suggested that serum UA (sUA) could be important in the pathogenesis and progression of renal diseases.^[10]^ Thus, numerous studies pointed at sUA as a potential therapeutic target for retarding the progression of hypertension and chronic kidney disease (CKD).^[11,12]^

Allopurinol and febuxostat are 2 xanthine oxidase inhibitors; they are commonly used for the long-term management of hyperuricemia in patients with gout because of their remarkable effects in decreasing the urate synthesis by impairing the conversion of hypoxanthine and xanthine to UA. In contrast with allopurinol, febuxostat is suggested to have a stronger hypouricemic effect without the danger of life-threatening hypersensitivity syndrome.^[13]^ Furthermore, the safety and efficacy of febuxostat have been proved without doses adjustment in patients with mild-to-moderate renal impairment.^[14,15]^

Numbers of studies suggested that allopurinol and febuxostat may be effective in decreasing blood pressure (BP) and retarding the progression of CKD. The possible mechanism of drug action involved may be related to the reduction of circulating sUA, improvements of the endothelial dysfunction and oxidative stress, and preventions of glomerular hypertension, afferent arteriolar thickening, and ischemic renal histologic changes.^[16,17]^ A previous randomized controlled trial (RCT) conducted among adolescents showed that allopurinol significantly decreased BP.^[1]^ Moreover, results from 2 meta-analyses also showed that allopurinol can significantly reduce BP in subjects with hyperuricemia.^[18,19]^ Generally speaking, previous studies mainly focus on the effects of allopurinol on BP. In recent years, a series of RCTs have been conducted to clarify whether febuxostat could also lower or influence the natural history of hypertension. However, the results of these RCTs remain controversial with some studies reported positive results; others found it had no significant effect. A meta-analysis would be appropriate to resolve the current controversy and reach a conclusive result.

Thus, this systematic review and meta-analysis will aim to evaluate the effect of febuxostat on BP in hyperuricemic patients. And such a study may find a new therapeutic option, that of control of a biochemical cause of hypertension.

## 2 Materials and methods

The current systematic review and meta-analysis will be reported following the Preferred Reporting Items for Systematic Reviews and Meta-Analyses (PRISMA).^[20]^ This review protocol is registered in the INPLASY register (registration number: INPLASY202050031).

### 2.1 Search strategy

**Figure 1:**
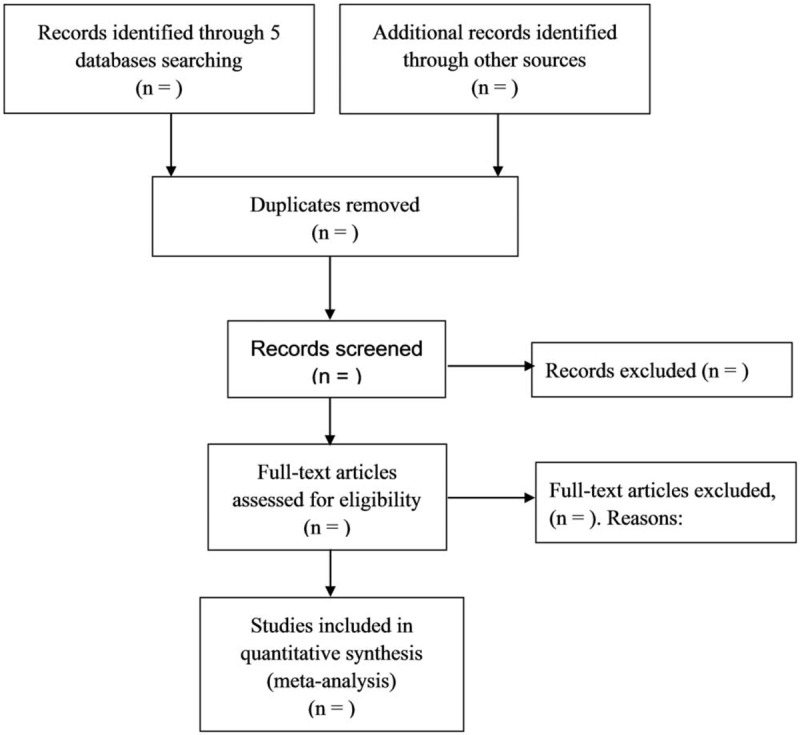
Flow diagram of study selection.

Databases including Medline, the Cochrane Library, EMBASE, Web of Science, and Cochrane Central Register of Controlled Trials (CENTRAL) will be searched. According to the PICOS principle, the search strategy will include the following terms: ((“Febuxostat”) AND (“hypertension” OR “hypertens∗” OR “blood pressure” OR “BP” OR “systolic blood pressure” OR “diastolic blood pressure” OR “SBP” OR “DBP”)). The ClinicalTrials.gov registry will be also searched for unpublished trials and authors will be contacted for additional information if necessary. Relevant references from included studies will be sought to retrieve additional eligible studies. No limits will be set on language, publication date, and type. Flow diagram of study selection will be in Figure 1.

### 2.2 Inclusion and exclusion criteria

The inclusion criteria will be as follows: RCTs with any follow-up duration and sample size; participants studied have a diagnosis of hyperuricemia or gout based on the preliminary or updated American College of Rheumatology (ACR) criteria; febuxostat should be applied in participants as an intervention at any dose; reported quantitative outcomes: the primary outcome will be BP, secondary outcomes will be sUA, serum creatinine, and estimated glomerular filtration rate (eGFR). The exclusion criteria will be as follows: non-RCTs; reports lacking relevant or sufficient data; duplicate literature.

### 2.3 Data extraction and management

Relevant data extraction will be performed by 2 researchers (JY and XYS) independently, and the third researcher (SMF) will be involved in a discussion for any disagreements. The following information of eligible articles will be extracted to a prepared data extraction form: author, year of publication, country of origin of the population studied, study design, sample size, duration, health status, mean age, number of males, doses of febuxostat, comorbid conditions, intervention, and outcomes. If raw data will not be directly provided in the text or tables, figures in the study would be referred to. Once relevant details will be insufficiently reported in studies, authors will be contacted by e-mails and the ClinicalTrials.gov register will be searched for further information.

### 2.4 Quality assessment

According to the Cochrane collaboration's update tool for assessing the risk of bias (Version 5.1.0),^[21]^ 2 reviewers (JY and XYS) will assess the quality of the studies independently, and the third researcher (SMF) will be consulted for any disagreements. The risk of bias will be classified as low, unclear, or high risk by evaluating the 7 components as random sequence generation, allocation concealment, blinding of outcome assessment, blinding of participants and personnel, incomplete outcome data, selective outcome reporting, and other bias. If necessary, we will try to e-mail the authors for extra information.

### 2.5 Statistical analysis

Stata 12.0 software will be used for statistical analysis. Dichotomous data will be expressed as the odds ratio (OR) with a 95% confidence interval (CI), and continuous data will be presented as the standardized mean difference (SMD) with 95% CI. *P* < .05 will be considered to indicate a statistically significant result.

### 2.6 Units of analysis issues

All parallel-designed studies will be included in this review. For cross-over trials, only the 1st treatment period data will be analyzed. For studies with multiple control groups, the unit of analysis will be used to each of all control groups.

### 2.7 Dealing with missing data

For insufficient or missing data, we will contact the authors by e-mail or phone as much as possible. Where data are unobtainable, we will assume that an event, without a reported outcome, has not occurred in participants and we will analyze only the available data. All analyses will be performed based on the intent-to-treat principle.

### 2.8 Assessment of heterogeneity

Heterogeneity will be tested by *χ*^2^-based Cochran Q statistic (*P* < .10 indicated statistically significant heterogeneity) and *I*^2^ statistic. If *I*^2^ < 50% and *P* > .1, a fixed-effects model will be used to pool the estimations across studies. If *I*^2^ ≥50% or P ≤ .1, after excluding clinical heterogeneity between studies, the random-effects model will be used.

### 2.9 Data synthesis

If there are sufficient studies and comparable outcomes, we will perform a meta-analysis. If not, we will perform a systematic review.

### 2.10 Subgroup analysis and investigation of heterogeneity

Subgroup analysis will be performed to explore the differences in the methodologic quality, race/ethnicity, sample size, and duration.

### 2.11 Sensitivity analysis

Sensitivity analysis will be used to observe changes in the pooled effect size and heterogeneity between included studies, to assess the reliability and stability of the pooled results.

### 2.12 Assessment of reporting biases

The funnel plot and Egger's and Begg's tests will be used to judge publication bias, and the trim and fill method will be used to correct the funnel asymmetry caused by publication bias.

### 2.13 Confidence in cumulative evidence

In this study, the level of evidence on all outcomes will be appraised by using an approach based on the Grading of Recommendations Assessment, Development, and Evaluation (GRADE). The quality of the body of evidence will be assessed based on 5 factors, including study limitations, effect consistency, imprecision, indirectness, and publication bias. The assessments will be categorized as high, moderate, low, and very low quality.

## 3 Discussion

The close relationship between hypertension and hyperuricemia is not a recent observation. Numerous studies have identified that increased UA was an independent risk factor for hypertension.^[9]^ Furthermore, scholars have launched a series of studies to identify whether UA-lowering agents might lower BP or impact the natural history of hypertension. To date, there are some RCTs conducted on patients concerning the antihypertensive efficacy of febuxostat have shown inconsistent results. On this basis, we will summarize the available evidence for aiming to evaluate the effect of febuxostat on BP in hyperuricemic patients. And such a study may find a new therapeutic option for hypertensive patients and assist clinicians and health professionals make clinical decisions.

## Author contributions

**Data analysis:** Simin Fan, Yang Gao.

**Data extraction:** Jia Yao, Xiaoyan Shi.

**Funding acquisition:** Qiu Chen.

**Methodology:** Qiu Chen.

**Project administration:** Qiu Chen.

**Resources:** Qiu Chen.

**Software:** Hengchang Hu, PanPan Wang.

**Writing – original draft:** Jia Yao, Xiaoyan Shi.

**Writing – review & editing:** Jia Yao, Xiaoyan Shi.
